# Characterization of Second-Order Mixing Effects in Reconstructed Cross-Spectra of Random Neural Fields

**DOI:** 10.1007/s10548-024-01040-8

**Published:** 2024-03-12

**Authors:** Rikkert Hindriks

**Affiliations:** Department of Mathematics, Faculty of Science, Vrije Universiteit Amsterdam, Amsterdam, The Netherlands

**Keywords:** Electroencephalography, Magnetoencephalography, Volume conduction, Signal leakage, Source modeling, Functional connectivity, Neural field

## Abstract

Functional connectivity in electroencephalography (EEG) and magnetoencephalography (MEG) data is commonly assessed by using measures that are insensitive to instantaneously interacting sources and as such would not give rise to false positive interactions caused by instantaneous mixing of true source signals (first-order mixing). Recent studies, however, have drawn attention to the fact that such measures are still susceptible to instantaneous mixing from *lagged* sources (i.e. second-order mixing) and that this can lead to a large number of false positive interactions. In this study we relate first- and second-order mixing effects on the cross-spectra of reconstructed source activity to the properties of the resolution operators that are used for the reconstruction. We derive two identities that relate first- and second-order mixing effects to the transformation properties of measurement and source configurations and exploit them to establish several basic properties of signal mixing. First, we provide a characterization of the configurations that are maximally and minimally sensitive to second-order mixing. It turns out that second-order mixing effects are maximal when the measurement locations are far apart and the sources coincide with the measurement locations. Second, we provide a description of second-order mixing effects in the vicinity of the measurement locations in terms of the local geometry of the point-spread functions of the resolution operator. Third, we derive a version of Lagrange’s identity for cross-talk functions that establishes the existence of a trade-off between the magnitude of first- and second-order mixing effects. It also shows that, whereas the magnitude of first-order mixing is determined by the inner product of cross-talk functions, the magnitude of second-order mixing is determined by a generalized cross-product of cross-talk functions (the wedge product) which leads to an intuitive geometric understanding of the trade-off. All results are derived within the general framework of random neural fields on cortical manifolds.

## Introduction

There are three basic ways in which spurious interactions between two measurement locations can arise in reconstructed source activity, namely through instantaneous linear mixing of incoherent, instantaneously coherent, or lagged coherent cortical activity. In the literature, different names have been used to describe these effects. In Drakesmith et al. (2013), the first two ways have been jointly referred to as *first-order artifacts* and the second way as *second-order artifacts*, in Palva and Palva (2012), the first way is referred to as *artificial synchrony* and the second and third ways are jointly referred to as *spurious synchrony*, and in Palva et al. (2018), the third way is referred to as *ghost interactions*. For the analysis in the current study, we find it useful to distinguish all three ways and will refer to them as zeroth-, first-, and second-order mixing effects, respectively. Thus, in this terminology, zeroth-, first-, and second-order effects refer to the effects of instantaneous linear mixing of incoherent, instantaneously coherent, and lagged coherent source activity, respectively, on the reconstruction of interactions in source space. These effects are generally different for different interaction measures (Palva et al. 2018).

Relatively few studies have focused on the relations between the effects of instantaneous linear mixing and the properties of the resolution operators that are used to reconstruct the source activity (Korhonen et al. 2014; Wens 2015; Farahibozorg et al. 2018; Ossadtchi et al. 2018; Wang et al. 2018; Hindriks 2020). This is perhaps surprising, because the way in which source signals are mixed is completely determined by the structure of the used resolution operator (Hauk et al. 2022; Hindriks 2020b) which is known and can therefore be exploited. For example, in Korhonen et al. (2014), Farahibozorg et al. (2018), resolution operators are exploited to design brain parcellations that minimize mixing of the reconstructed source signals and in (Wens 2015), Ossadtchi et al. (2018), Hindriks (2020), they are used to construct interaction measures that are insensitive to zeroth- and first-order mixing effects, whereas still being sensitive to instantaneous interactions. Indeed, classical interaction measures that do not make use of the resolution operator, such as the imaginary coherence (Nolte et al. 2004), the (weighted) phase-lag index (Stam et al. 2007; Vinck et al. 2011), the imaginary phase-locking value (Palva and Palva 2012), and the lagged coherence (Pascual-Marqui 2007), are insensitive to zeroth- and first-order mixing effects, but are per construction insensitive to instantaneous interactions. It thus seems that studying the relation between mixing effects and resolution operators can provide new insights into and methods for the analysis of functional brain connectivity.

This motivates the current study, which aims to clarify some basic relations between the properties of resolution operators and the effects of linear mixing on the reconstruction of functional interactions. We focus on second-order effects because they are the least well studied and because it has recently come to light that they can cause large numbers of false positives in EEG/MEG functional connectivity analysis, even when using classical interaction measures (Palva et al. 2018). Furthermore, we focus on second-order effects on the imaginary part of the cross-spectral function of cortical activity, since this is the simplest interaction measure that is insensitive to zeroth- and first-order mixing. The analysis of second-order effects on normalized interaction measures such as the phase-lag index and the lagged coherence is much more complicated due to their highly non-linear behavior and will be left for a future study. We do note, however, that normalization is not strictly necessary when the measures are used as test-statistics in a hypothesis test for significant interaction. This is because normalization only serves to obtain measures whose null-distribution is independent of the other model parameters (e.g. the variances of the signals) but does not change the conclusion of the test.

In our analysis, we adopt a spatially continuous description of cortical activity, because it is physically the most realistic (Bresslof 2012), more general than a discrete description (the latter is a special case of the former), and it is more natural when the aim is to obtain a basic understanding of the phenomena. Furthermore, since in EEG/MEG studies the interest is often in oscillatory activity, we model cortical activity in the frequency domain. Thus, at any point in time, cortical activity is described by a complex-valued scalar field on the cortical manifold, whose values are the time-frequency coefficients of the activity at a fixed frequency. The scalar field is treated as random and hence cortical activity is modeled by a (zero-mean) random scalar field on the cortical manifold. We note that if the field has a Gaussian probability distribution, its statistics are completely described by its cross-spectral function, and hence our characterization of mixing effects is complete. If the field is non-Gaussian, however, a complete characterization of mixing effects needs to take into account higher-order statistics. We leave this for a future study.

The mapping from true fields to their reconstructions is modeled by a linear integral operator with a real-valued kernel, which models the composition of a linear forward operator and a linear inverse operator and is a generalization of the resolution operator to continues space (Hauk et al. 2022). The real-valuedness of the reconstruction kernel reflects the instantaneous nature of the forward mapping (Hämäläinen et al. 1993). This property is crucial in the analysis of functional connectivity in source space and the classical interaction measures are insensitive to zeroth- and first-order mixing precisely because of this property.

We first characterize the effects of zeroth-, first-, and second-order mixing in terms of the cross-talk functions of the reconstruction kernel and derive alternative representations of the first- and second-order effects in terms of the symmetry properties of configurations of measurement and source locations. The representation of the second-order effects will form the basis for the subsequent analyses. In this representation, the contribution of a lagged interaction between cortical activity at a given pair of locations to the reconstructed lagged interaction between another pair of locations is proportional to a suitably defined notion of symmetry of the configuration under interchanging the measurement locations *x* and $x'$.

This representation is the generalization of that described in Ossadtchi et al. (2018), Hindriks (2020) to continuous space. We use it to characterize the configurations with maximal and minimal second-order effects, to obtain a local approximation of second-order mixing effects in terms of the curvature of the cross-talk functions, to show that second-order effects are not limited to regions surroundings the measurement locations, and to establish the existence of trade-off between zeroth- and first-order effects. This trade-off is described in the form of Lagrange’s identity for pairs of cross-talk functions and relates the magnitudes of the zeroth- and second-order effects. We also provide a geometric interpretation of this identity in which the magnitude of second-order leakage between a given pair of locations is identified with the surface area of the parallelogram spanned by the two cross-talk functions and the magnitude of zeroth-order effects can be identified with their inner product. This provides a direct geometric intuition for the existence of this trade-off.

## Materials and Methods

### Random Neural Fields

We model cortical activity in the frequency domain by a zero-mean stationary Gaussian random field on the cortical manifold $\Omega$. The frequency coefficient of the field at a location $x\in \Omega$ is denoted by $s(x)\in \mathbb {C}$ and is considered to be a random variable. Stationarity and Gaussianity together imply that the field is completely described by its cross-spectral function

1 $$\begin{aligned} \gamma (x,x') = \mathbb {E}\left[ s(x)s(x')^*\right] , \end{aligned}$$

for all $x,x'\in \Omega$, where the superscript $*$ denotes the complex-conjugate and $\mathbb {E}$ denotes expectation over temporal windows (i.e. in the case of ongoing activity) of over trials (i.e. in the case of induced activity). Note that the cross-spectral function is conjugate-symmetric: $\gamma (x',x) = \gamma (x,x')^*$ for all $x,x'\in \Omega$ so that $\text {Re}(\gamma (x',x)) = \text {Re}(\gamma (x,x'))$ and $\text {Im}(\gamma (x',x)) = -\text {Im}(\gamma (x,x'))$. Also, $\gamma (x,x)\ge 0$ is the power of the cortical activity at location *x*, which we will denote by $\sigma ^2(x)$.

Following the terminology used in the field of spatial statistics (VanMarcke 1983), a neural field is called *homogeneous* if $\gamma (x,x') = \gamma (d(x,x'))$ for all $x,x'\in \Omega$, where $d(x,x')$ is a distance measure on the cortical manifold (e.g. the geodesic distance). In particular, a homogeneous field has constant power: $\sigma ^2(x) = \sigma ^2$ for all $x\in \Omega$. A neural field is *incoherent* if $\gamma (x,x') = \sigma ^2(x)\delta (x-x')$ for all $x,x'\in \Omega$, where $\delta$ denotes the Dirac delta function. A neural field is *coherent* if $|\gamma (x,x')|$ is constant, where the vertical bars denote the modulus. A neural field can be represented as $s(x,t) = \alpha (x,t)\exp {(i\phi (x,t))}$, where $\alpha (x,t)$ and $\phi (x,t)$ are the associated *amplitude field* and *phase field*, respectively.

### Linear Instantaneous Mixing of Neural Fields

When a linear inverse operator is used to reconstruct a neural field *s*, either based on observed electric potentials (EEG and ECoG) or on magnetic fluxes outside the head (MEG), the reconstructed field $\hat{s}$ is related to the true field by

2 $$\begin{aligned} \hat{s}(x) = {\mathop {\int }\limits _{\Omega }} G(x,y)s(y), \end{aligned}$$

where *G*(*x*, *y*) is the *resolution kernel*. The mapping from *s* to $\hat{s}$ is the concatenation of a linear forward operator and a linear inverse operator, both of which are left implicit here. The forward operator describes how the neural field is mapped to the sensors and in practice is obtained by numerically solving the quasi-static Maxwell equations (Hämäläinen et al. 1993; Mosher et al. 1999). The inverse operator can be non-adaptive such as the minimum norm operator (Grech et al. 2008) or adaptive such as a beamformer (Hillebr and Barnes 2005). The resolution kernel describes how the neural field *s* is *mixed* to obtain its reconstruction $\hat{s}$. Equation (2) shows that mixing is linear and instantaneous, the latter is true because the resolution kernel is real-valued.

The resolution kernel assigns to every pair of cortical locations $x,y\in \Omega$ a real number *G*(*x*, *y*) that determines how strong the true field at *y* contributes to the reconstructed field at *x*. In particular, the diagonal of the resolution kernel, i.e. the mapping $(x,x)\mapsto G(x,x)$ determines the gain of the reconstructed field at *x*. For $y\ne x$, this can be considered to be the amount of "leakage" from *y* to *x*. In these terms, the well-known surface bias of linear inverse operators is reflected in a low gain in cortical sulci and subcortical structures and a high gain in locations that are closer to the sensors (Grech et al. 2008). One class of resolution kernels are obtained by assuming that the leakage from *y* to *x* only depends on the (Euclidean) distance (Table 1) $||x-y||$ between *y* and *x*. This corresponds to modeling the kernel as $G(x,y) = f(||x-y||)$ for some function *f*. Typically, *f* decreases with increasing distance, for instance $f(||x-y||) = 1/(1 + ||x-y||)$ or $f(||x-y||) = \text {exp}(-||x-y||^2)$.

**Table 1:** Listed are quantities, their symbols, and meanings. Their formal definitions can be found in the main text

Symbol	Meaning
$\Omega$	Cortical manifold
*s*(*x*)	Random neural field
$\gamma (x,x')$	Fields’ cross-spectrum
$\sigma ^2(x)$	Field power
$G(x,x')$	Resolution kernel
$g_x(y)$	Cross-talk function at *x*
$S_+(x,x',y,z)$	Sensitivity to first-order leakage
$S_-(x,x',y,z)$	Sensitivity to second-order leakage

The variables $x,x',y$ and *z* denote arbitrary locations on the cortical manifold $\Omega$

**Fig. 1:**
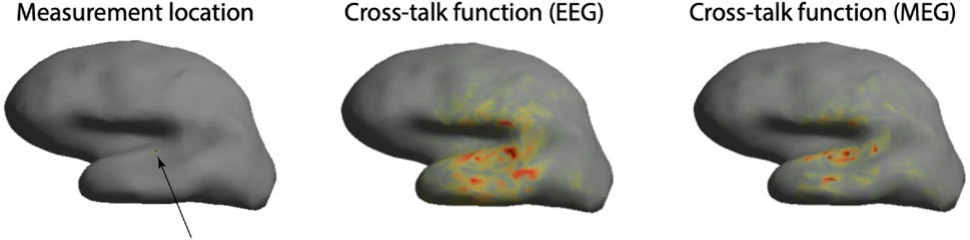
*EEG and MEG cross-talk functions.* Shown is an inflated cortical surface with a measurement location in the left superior temporal lobe (left panel), together with the associated (absolute values of the) cross-talk functions of the minimum norm resolution operator for EEG (middle panel) and MEG (right panel) forward models. In calculating the resolution operators, the regularization level was set to $10^{-8}$

**Fig. 2:**
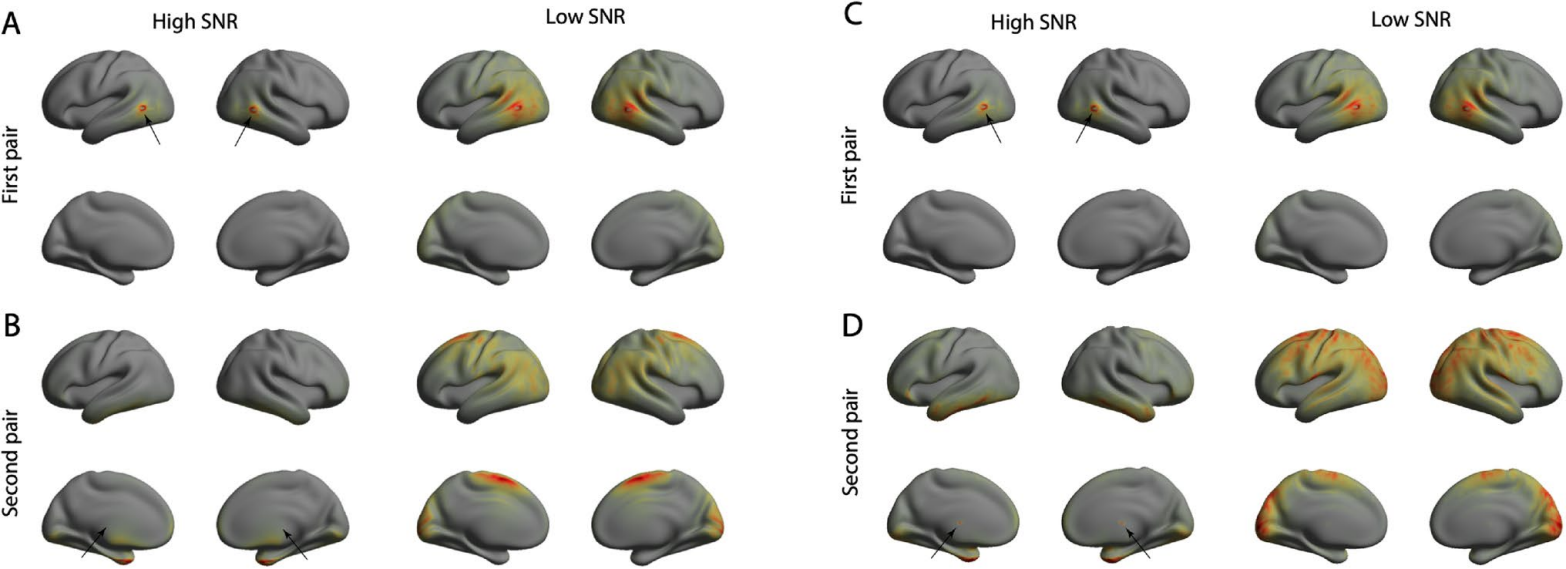
*First- and second-order leakage in MEG minimum norm solution.* **A** Total first-order leakage into the homologue measurement pair located in the posterior temporal lobe (black arrows) in the high SNR scenario. **B** Total first-order leakage into the connectivity of the homologue measurement pair located in the medial wall (black arrows) in the high SNR case. **C** Same format as **A**. but for second-order leakage. **D** Same format as **B**. but for second-order leakage

When viewed as a function of *x* for fixed *y*, *G*(*x*, *y*) is referred to as the *point-spread function* of the kernel at *y* and when viewed as a function of *y* for fixed *x*, *G*(*x*, *y*) is referred to as *cross-talk function* of the kernel at *x*. This terminology follows that used in the discrete case (Hauk et al. 2022). If the resolution operator is symmetric, i.e. $G(y,x) = G(x,y)$, the point-spread functions are equal to the cross-talk functions. The cross-talk functions will play a central role in this study and we will denote them by $g_x(y)$. Since cross-talk functions can be added and multiplied by real-valued scalars, they form an infinite-dimensional vector space over $\mathbb {R}$. We define an inner product on this vector space by

3 $$\begin{aligned} \left<g_x,g_{x'}\right>= {\mathop {\int }\limits _{\Omega }} g_x(y)g_{x'}(y), \end{aligned}$$

and denote the associated norm by $||g_x|| = \sqrt{\left<g_x,g_x\right>}.$ Since $\Omega$ is compact, this turns the vector space into a Hilbert space over $\mathbb {R}$.

Fig. 1 shows a measurement location on the cortical surface (left superior temporal lobe), together with the cross-talk functions of the minimum norm resolution operator for both EEG and MEG forward models. The cortical surface and forward models were provided by the MNE Software (Gramfort et al. 2015). The MEG scanner was a 306-channel Elekta-Neuromag vectorview (MEGIN) system and the EEG cap had 60 electrodes. Observe that cross-talk is high around the measurement locations and decreases for larger distances and that the MEG cross-talk function is more localized than the EEG cross-talk function. Also observe that the cross-talk functions lack smoothness. This is caused by the convolutions of the cortex surrounding the measurement location and which are not visible on the inflated cortex. The figure shows that the above examples of resolution operators are highly idealized.

### Cross-Spectral Functions of Reconstructed Fields

The cross-spectral function $\hat{\gamma }$ of a reconstructed random neural field is related to the cross-spectral function of the true field by

4 $$\begin{aligned} \hat{\gamma }(x,x') = {\mathop {\int }\limits _{\Omega \times \Omega }} G(x,y)G(x',z)\gamma (y,z), \end{aligned}$$

where $\times$ in $\Omega \times \Omega$ denotes the Cartesian product of $\Omega$ with itself. Below, we will refer to $\hat{\gamma }$ simply as the reconstructed cross-spectral function. The non-negative definiteness of the reconstructed cross-spectral function follows directly from that of the true cross-spectral function. The reconstructed cross-spectral function can be decomposed as

5 $$\begin{aligned} \hat{\gamma }(x,x') = {\mathop {\int }\limits _{\Omega }} g_x(u)g_{x'}(u)\sigma ^2(u) + {\mathop {\int }\limits _{\Omega \times \Omega }}g_x(y)g_{x'}(z)\text {Re}(\gamma (y,z)) + i{\mathop {\int }\limits _{\Omega \times \Omega }}g_x(y)g_{x'}(z)\text {Im}(\gamma (y,z)), \end{aligned}$$

where $i=\sqrt{-1}$ denotes the imaginary unit. We refer to the terms on the right-hand-side as zeroth-, first-, and second-order terms, respectively. Note that the zeroth-order term is independent of the interactions in the true field as described by $\gamma (y,z)$ with $z\ne y$, and only depends on its power $\sigma ^2(u)$ at different cortical locations $u\in \Omega$. The first- and second-order terms, on the other hand, only depend on the instantaneous (i.e. real) and lagged (i.e. imaginary) interactions of the true field, respectively, and are independent of its power.

The above decomposition emphasizes a basic property of random neural fields and their reconstructions, which is that instantaneous interactions can only give rise to instantaneous interactions in the reconstructed field and the same holds for lagged interactions. This implies that if a reconstructed cross-spectral function has non-vanishing imaginary part, the true field must exhibit lagged interactions. It is this basic property that enables the construction of interaction measures that are insensitive to first- and second-order mixing.

If the true field is incoherent, i.e. $\gamma (x,x') = \sigma ^2(x)\delta (x-x')$, Eq. (5) only has a zeroth-order term:

$$\begin{aligned} \hat{\gamma }(x,x') = {\mathop {\int }\limits _{\Omega }}g_x(u)g_{x'}(u)\sigma ^2(u). \end{aligned}$$

If the field is also homogeneous, i.e. $\sigma ^2(x) = \sigma ^2$, the zeroth-order term reduces to

$$\begin{aligned} \hat{\gamma }(x,x') = \sigma ^2{\mathop {\int }\limits _{\Omega }} g_x(u)g_{x'}(u) = \sigma ^2\left<g_x,g_{x'}\right>, \end{aligned}$$

and, in particular, the reconstructed power is $\hat{\sigma }^2(x) = ||g_x||^2\sigma ^2$. This direct relation between the zeroth-order effect of mixing and the inner product between the cross-talk functions is a well-known property of linear inverse operators.

In Sect. Relation between total zeroth- and second-order mixing effects we characterize the relation between zeroth- and second-order effects and the cross-talk functions and will see that this involves a generalization of the cross-product between the cross-talk functions known as the wedge product. To obtain this characterization, it will be convenient to first derive a different representation of the reconstructed cross-spectral function, which will be done in Sect. Basic identities for first- and second-order mixing.

### Basic Identities for First- and Second-Order Mixing

The conjugate symmetry of the cross-spectral function can be used to obtain formulas for the real and imaginary parts of the reconstructed cross-spectral function that simplify the analyses of mixing effects in the subsequent sections. Specifically, in Appendix A, we derive two identities that express the real/imaginary part of the reconstructed cross-spectral function in terms of the real/imaginary part of the true cross-spectral function. For $x\ne x'$ these identities are:

6 $$\begin{aligned} \text {Re}(\hat{\gamma }(x,x')) = {\mathop {\int }\limits _{A}}S_+(x,x',y,z)\text {Re}(\gamma (y,z)), \end{aligned}$$

and

7 $$\begin{aligned} \text {Im}(\hat{\gamma }(x,x')) = {\mathop {\int }\limits _{A}} S_-(x,x',y,z)\text {Im}(\gamma (y,z)), \end{aligned}$$

where the integral is taken over the set $A = \{(y_1,y_2,z_1,z_2) | y_1 > z_1\}$. The functions $S_+$ and $S_-$ are defined by

$$\begin{aligned} S_+(x,x',y,z) = g_x(y)g_{x'}(z) + g_{x'}(y)g_x(z), \end{aligned}$$

and

$$\begin{aligned} S_-(x,x',y,z) = g_x(y)g_{x'}(z) - g_{x'}(y)g_x(z). \end{aligned}$$

We will refer to an ordered quadruple $(x,x',y,z)$, in which *x* and $x'$ are measurement locations and *y* and *z* are source locations, as a *configuration*. The squared values $S^2_+(x,x',y,z)$ and $S^2_-(x,x',y,z)$ can be interpreted as measures of the lack of anti-symmetry and symmetry, respectively, of the configuration $(x,x',y,z)$, under interchanging the measurement locations *x* and $x'$. Symmetry in this context does not refer to spatial symmetry, but to the more abstract notion of invariance of an object under a class of transformations. Thus, the lack of symmetry and anti-symmetry of a configuration are directly related to the mixing effects and both are determined by the quantities $g_x(y)$, $g_{x'}(z)$, $g_x(z)$, and $g_{x'}(y)$.

Eq. (6) shows that the contribution of a true instantaneous interaction $\text {Re}(\gamma (y,z))$ between *y* and *z* to the reconstructed instantaneous interaction $\text {Re}(\hat{\gamma }(x,x'))$ between *x* and $x'$ is proportional to the lack of anti-symmetry of the configuration $(x,x',y,z)$. Likewise, Eq. (7) shows that the contribution of a true lagged interaction between *y* and *z* to the reconstructed lagged interaction between *x* and $x'$ is proportional to the lack of symmetry of the configuration $(x,x',y,z)$. In other words, $S_+(x,x',y,z)^2$ and $S_-(x,x',y,z)^2$ are measures for the strength of, respectively, first- and second-order mixing of the true interaction between *y* and *z* into the reconstructed interaction between *x* and $x'$.

Note that $S_+(x,x',y,z) = 0$ precisely when $g_x(y)g_{x'}(z) = -g_{x'}(y)g_x(z),$ i.e. when $g_x(y)g_{x'}(z)$ flips sign under the transformation that interchanges *x* and $x'$. We refer to such a configuration as *anti-symmetric*. Likewise, $S_-(x,x',y,z) = 0$ precisely when $g_x(y)g_{x'}(z) = g_{x'}(y)g_x(z),$ i.e. when $g_x(y)g_{x'}(z)$ in invariant under the transformation that interchanges *x* and $x'$. We refer to such a configuration as *symmetric*. Thus, symmetric/anti-symmetric configurations are those for which second-order/first-order mixing effects are absent. By summing $S^2_-(x,x',y,z)$ and $S^2_+(x,x',y,z)$ we find that they satisfy

8 $$\begin{aligned} S_+^2(x,x,y,z) + S_-^2(x,x',y,z) = 2g_x^2(y)g_{x'}^2(z) + 2g_{x'}^2(y)g_x^2(z), \end{aligned}$$

which holds for all configurations $(x,x',y,z)$. The term on the right-hand-side of Eq. (8) is a measure for the total (i.e. first- and second-order) strength of mixing of the true interaction between *y* and *z* into the reconstructed interaction between *x* and $x'$ and hence shows that, given the total mixing strength, there is a trade-off between the strength of first- and second-order mixing. In particular, given the total mixing strength, symmetric configurations have maximal first-order mixing effects and anti-symmetric configurations have maximal second-order mixing effects.

## Results

### Illustration of the Basic Identities

As an illustration of the basic identities, we selected two pairs of homologue measurement locations $(x,x')$ on the cortical surface and, for each of the pairs, computed $S_+^2$ and $S_-^2$ for all homologue source pairs (*y*, *z*) obtained from the cross-talk functions of the minimum norm resolution operator. The forward operators (i.e. leadfield matrices) were obtained from 89 subjects of the MEG data-set provided by the Human Connectome Project (HCP) (Larson-Prior et al. 2013) using the Fieldtrip toolbox (Oostenveld et al. 2011). The resulting values were averaged over all subjects. In calculating the resolution operators, we used two different values for the regularization level: $\lambda = 10^{-10}$ (high SNR) and $\lambda = 10^{-6}$ (low SNR).

Fig. 2A shows the first-order leakage in both conditions (high and low SNR) for the first pair of locations (black arrows). It shows that leakage is more severe when the SNR is low, but that it is still confined to the tissue surrounding the measurement locations. The same holds for second-order leakage (Fig. 2C). Figure 2B shows the first-order leakage in both conditions for the second pair of locations (black arrows). The figures shows that, in both conditions, leakage is not confined anymore to a neighborhood of the measurement locations and that distant cortical regions leak into the connectivity between the measurement locations. Figure 1D shows that the same is true for second-order leakage. As a reference, the optimal regularization level for resting-state MEG data in the alpha frequency band (7–13 Hz) from this data-set, as determined by generalized cross-validation, is about $\lambda = 10^{-8.5}$(Hindriks et al. 2017).

### Configurations with Maximal and Minimal Mixing Effects

To illustrate the use of the basic identities (Eqs. (6) and (7)) we provide characterizations of the configurations that are maximally and minimally sensitive to first- and second-order mixing effects. These characterizations will be in terms of the configurations’ geometry, i.e. in terms of the relative positions of the measurement and source locations. Because these positions determine the symmetry of the configuration only indirectly via the cross-talk functions, to relate the symmetry to the geometry of a configuration, we need to make some mild assumptions about the cross-talk functions. We assume that for every location *x*, (i) $g_x$ is maximal in *x*, (ii) $g_x$ decreases with increasing Euclidean distance from *x*, and (iii) $g_x$ is non-negative, i.e. $g_x\ge 0$.

We ask which configurations $(x,x',y,z)$ minimize/maximize $S_-(x,x',y,z)^2$ and $S_+(x,x',y,z)^2$. The only non-trivial case is for which configurations $S_-(x,x',y,z)^2$ is minimized and will be discussed last. First consider for which configurations

$$\begin{aligned} S_-(x,x',y,z)^2 = (g_x(y)g_{x'}(z)-g_{x'}(y)g_x(z))^2, \end{aligned}$$

is maximized. This is the case if $g_x(y)g_{x'}(z)$ is maximal and $g_{x'}(y)g_x(z)$ is minimal (or the other way around). Now, $g_x(y)g_{x'}(z)$ is maximal if $y=x$ and $z=x'$ (assumption (i)), in which case $g_{x'}(y)g_x(z)$ reduces to $g_{x'}(x)g_x(x')$, which is minimal if *x* and $x'$ are far apart (assumption (ii)). Thus, the configurations that are maximally sensitive to second-order mixing effects are those for which the source locations coincide with the measurement locations and for which the measurement locations are far apart. Now consider for which configurations

$$\begin{aligned} S_+(x,x',y,z)^2 = (g_x(y)g_{x'}(z)+g_{x'}(y)g_x(z))^2, \end{aligned}$$

is maximized. This is the case if both $g_x(y)g_{x'}(z)$ and $g_{x'}(y)g_x(z)$ are maximal. The first term is maximal if $y=x$ and $z=x'$ (assumption (i)). This reduces the second term to $g_{x'}(x)g_x(x')$, which is maximal if $x=x'$ (assumption (i)). Thus, the configurations that are maximally sensitive to first-order mixing effects are those in which both measurement and source locations coincide. From the above expression for $S_+(x,x',y,z)^2$ it is also immediately clear that $S_+(x,x',y,z)^2$ is minimal if both terms $g_x(y)g_{x'}(z)$ and $g_{x'}(y)g_x(z)$ are zero, which is the case if one of the cross-talk functions in each term is zero, for which there are four possibilities. Two of these are that both sources are sufficiently far from both measurement locations (assumption (i)).

We are left with the question for which configurations is $S_-(x,x',y,z)^2$ is minimal, or equivalently, for which configurations $g_x(y)g_{x'}(z) = g_{x'}(y)g_x(z).$ There is no general answer to this question, because it depends on the particular form of the cross-talk functions $g_x$ and $g_{x'}$. Consider the special case

$$\begin{aligned} g_x(y) = \exp {(-||x-y||^2/2\kappa ^2)}, \end{aligned}$$

where $||x-y||$ denotes the Euclidean distance between *x* and *y* and $\kappa$ is the characteristic scale of $g_x$. The above condition then takes the form

$$\begin{aligned} ||x-y||^2 + ||x'-z||^2 = ||x'-y||^2 + ||x-z||^2, \end{aligned}$$

which is equivalent to $\left<x-x',y-z \right>= 0,$ where the brackets denote the dot product in $\mathbb {R}^3$. This shows that sensitivity to lagged interactions is zero precisely when the line through the measurement locations *x* and $x'$ and the line through the source locations *y* an *z* are perpendicular. In particular, for fixed source locations *y* and *z*, the measurement locations for which sensitivity to lagged interactions is zero form a plane in $\mathbb {R}^3$. If the configuration is confined to a plane, they form a line in $\mathbb {R}^2$, and in the configuration is confined to a line, sensitivity to lagged interactions is never zero.

In general, characterizations of the symmetric configurations are more complicated than in the above special case. For example, if $g_x(y)$ is a rational function (i.e. a quotient of polynomials) in $||x-y||^2$, the planes will be replaced by curved surfaces. An example is

$$\begin{aligned} g_x(y) = \frac{1}{1 + ||x-y||^2}, \end{aligned}$$

in which case the symmetric configurations are characterized by the condition

$$\begin{aligned} (1 + ||x-y||^2)(1 + ||x'-z||^2) - (1 + ||x'-y||^2)(1 + ||x-z||^2) = 0. \end{aligned}$$

For fixed source locations *y* and *z*, the solutions of this equation form a two-dimensional surface in $\mathbb {R}^3$.

### Mixing Effects in the Vicinity of the Measurement Locations

In Sect. 3.2 we established that the configurations that are maximally sensitive to second-order mixing effects are those for which the source locations coincide with the measurement locations and for which the measurement locations are far apart. We now consider the case that the sources are located in the vicinity of the measurement locations (and the latter are far apart). This case was explored extensively using numerical simulations by Palva et al. (2018). Specifically, we relate $S_-(x,x',y,z)$ to the geometric properties of the cross-talk functions $g_x$ and $g_{x'}$ in the vicinity of *x* and $x'$, respectively.

If *x* and $x'$ are far apart, we neglect the term $g_{x'}(x)g_x(x')$ in $S_-(x,x',y,z)$ and approximate $g_x(y)$ and $g_{x'}(z)$ by a second-order Taylor series in *x* and $x'$, respectively. For *y* close to *x* and *z* close to $x'$, $S_-(x,x',y,z)$ can then be approximated as

9 $$\begin{aligned} S_-(x,x',y,z) = g_x(x)g_{x'}(x') + \frac{1}{2}(y-x)^\textrm{T}H_g(x)(y-x)g_{x'}(x') + \frac{1}{2}(z-x')^\textrm{T}H_g(x')(z-x')g_{x}(x) \end{aligned}$$

where $H_g(x)$ and $H_g(x')$ denote the Hessian matrices of $g_x$ at *x* and of $g_{x'}$ at $x'$, respectively (see Appendix B). Thus, the (*i*, *j*)-th entry of $H_g(x)$ is given by the second-order partial derivative of $g_x$ to $x_i$ and $x_j$ at *x*:

$$\begin{aligned} \left( H_g(x)\right) _{i,j} = \frac{\partial ^2 g_x(x)}{\partial x_ix_j}. \end{aligned}$$

This approximation is also valid for $S_+(x,x',y,z)$. Equation (9) shows that the local structure of signal leakage is determined by the Hessian matrices of the cross-talk functions at the two measurement locations and that the effects are additive. Assumptions (i) and (ii) imply that $H_g(x)$ and $H_g(x')$ are negative definite. Combining this with assumption (iii) we conclude that the second and third term on the right-hand-side of Eq. (9) are negative. From this we can conclude that leakage is maximal at the measurement locations and decreases in the neighborhood of the measurement locations.

As a special case, suppose that both Hessian matrices are proportional to the $3\times 3$ identity matrix, with proportionality constant $-\xi$ for some $\xi >0$, i.e. $H_g(x) = H_g(x') = -\xi I_3$, where $I_3$ denotes the $3\times 3$ identity matrix. Then Eq. (9) reduces to

10 $$\begin{aligned} S_-(x,x',y,z) = g_x(x)g_{x'}(x') - \frac{\xi }{2}\left( ||y-x||^2g_{x'}(x') + {2}||z-x'||^2g_{x}(x)\right) . \end{aligned}$$

Since the Gaussian curvature *K* of $g_x$ at *x* and of $g_{x'}$ at $x'$ is given by the determinant of the respective Hessian matrices, which equals $\xi ^3$, we see that the local leakage effect is proportional to $K^{1/3}$. If, in addition, $g_x(x)=g_{x'}(x') = 1$ and $||y-x|| = ||z-x||=\delta$, we obtain the following approximation:

11 $$\begin{aligned} S_- \approx 1 - \xi \delta ^2, \end{aligned}$$

which is also valid for $S_+$. It shows that, in the vicinity of the measurement locations, leakage decreases linearly with $\xi$ and quadratically with distance. As an example, let $g_u(v) = \exp {(-||u-v||^2/2\kappa ^2)}$, where $\kappa >0$ is the characteristic width of $g_u$. Thus, large values of $\kappa$ correspond to a low spatial resolution. Its Hessian is proportional to the $3\times 3$ identity matrix with $\xi = 1/\kappa ^2$ so that $S_-\approx 1-\delta ^2/\kappa ^2.$

### Relation Between Total Zeroth- and Second-Order Mixing Effects

In Sect. 2.4 we showed that, for a given configuration of measurement and source locations, and given the total mixing strength, there is a tradeff between first- and second-order mixing effects. We now show that a related trade-off exists when integrated over all true interactions.

As a measure for the total strength of zeroth-order mixing effects in the reconstruction of the interaction between *x* and $x'$ we take the squared inner product of the cross-talk functions at *x* and $x'$:

12 $$\begin{aligned} \left<g_x,g_{x'}\right>^2 = \left( {\mathop {\int }\limits _{\Omega }} g_x(u)g_{x'}(u)\right) ^2. \end{aligned}$$

Note that zeroth-order effects are absent precisely when $g_x$ and $g_{x'}$ are orthogonal. As a measure for the total strength of second-order mixing we take the quantity $||g_x\wedge g_{x'}||^2$, which we define by

$$\begin{aligned} ||g_x\wedge g_{x'}||^2 = \int _{A}S_-(x,x',y,z)^2. \end{aligned}$$

The notation $||g_x\wedge g_{x'}||$ will be explained in Sect. 3.5 when we discuss the special case of finitely many point-sources. We first discuss the following relation, which is derived in Appendix B:

13 $$\begin{aligned} ||g_x\wedge g_{x'}||^2 + \left<g_x,g_{x'}\right>^2 = ||g_x||^2 ||g_{x'}||^2, \end{aligned}$$

where $||g_x||$ denotes the norm of $g_x$ (see Sect. 2.2). It is a continuous version of Lagrange’s identity and shows that, given the norms $||g_x||$ and $||g_{x'}||$ of the cross-talk functions, there is a trade-off between the total zeroth- and second-order mixing effects to the reconstruction of the interaction between *x* and $x'$.

Note that, given $||g_x||$ and $||g_{x'}||$, if $g_x$ and $g_{x'}$ are orthogonal, zeroth-order effects are absent and second-order effects are maximal and if $g_x$ and $g_{x'}$ are linearly dependent, second-order effects are absent and zeroth-order effects are maximal. By dividing both sides of Eq. (13) by $||g_x||^2 ||g_{x'}||^2$, we obtain the following relation

14 $$\begin{aligned} \frac{||g_x\wedge g_{x'}||^2}{||g_x||^2 ||g_{x'}||^2} + \frac{\left<g_x,g_{x'}\right>^2}{||g_x||^2 ||g_{x'}||^2} = 1, \end{aligned}$$

between the normalized measures of the strengths of zeroth- and second-order mixing effects. This relation can also be written as

15 $$\begin{aligned} \sin ^2{\alpha } + \cos ^2{\alpha } = 1, \end{aligned}$$

where $\alpha$ denotes the angle between $g_x$ and $g_{x'}$ and clearly shows the trade-off between the relative strengths of zeroth- and second-order mixing effects.

Lastly, we provide a geometric interpretation of the trade-off in Eq. (14) in terms of the parallelogram spanned by $g_x$ and $g_{x'}$. For two finite-dimensional vectors *v* and $v'$, the area of the parallelogram spanned by *v* and $v'$ equals the square root of the determinant of their Gram matrix:

$$\begin{aligned} \text {area}^2 = \text {det} \begin{bmatrix} ||v||^2 &{} \left<v,v'\right>\\ \left<v',v\right>&{} ||v'||^2 \end{bmatrix} = ||v||^2 ||v'||^2 - \left<v,v'\right>^2. \end{aligned}$$

This can be generalized to infinite-dimensional Hilbert spaces, which allows to define the area of the parallelogram spanned by two cross-talk functions. Thus, the area of the parallelogram spanned by two cross-talk functions $g_x$ and $g_{x'}$ can be defined as

16 $$\begin{aligned} \text {area}^2 = \text {det} \begin{bmatrix} ||g_x||^2 &{} \left<g_x,g_{x'}\right>\\ \left<g_{x'},g_x\right>&{} ||g_{x'}||^2 \end{bmatrix} = ||g_x||^2 ||g_{x'}||^2 - \left<g_x,g_{x'}\right>^2. \end{aligned}$$

Note that the area is always non-negative because the Gram matrix is non-negative definite. Comparing Eq. (16) with Eq. (13) we conclude that

17 $$\begin{aligned} ||g_x\wedge g_{x'}|| = \text {area}, \end{aligned}$$

which shows that the total strength of second-order mixing to the reconstructed interaction between *x* and $x'$ can be interpreted as the area of the parallelogram spanned by the cross-talk functions $g_x$ and $g_{x'}$.

### The Discrete Case

In this section we consider the special case that the neural field comprises *N* point-sources at locations $x_1,\dots ,x_N\in \Omega$. This largely amounts to replacing integrals by sums, but will also provide some geometric insight into the structure of mixing effects. The neural field in this case takes the following form:

$$\begin{aligned} s(x) = \sum _{n=1}^N\delta (x-x_n)s_n, \end{aligned}$$

where $s_n$ is the Fourier coefficient of the *n*-th source. Let $\gamma _{n,m} = \mathbb {E}\left[ s_ns_m^*\right]$ be the cross-spectrum between the *n*-th and *m*-th source. The cross-spectrum $\gamma (x,x')$ between measurement locations *x* and $x'$ now becomes

$$\begin{aligned} \gamma (x,x') = \sum _{n=1}^N\sum _{m=1}^N\delta (x-x_n)\delta (x-x_m)\gamma _{n,m}. \end{aligned}$$

Furthermore, the reconstructed field takes the form

$$\begin{aligned} \hat{s}(x,t) = \sum _{n=1}^Ng_x(x_n)s_n, \end{aligned}$$

where $g_x$ is the cross-talk function at *x*. We assume that the measurement locations do not coincide with the source locations so that the reconstructed field is entirely spurious. Note that the cross-talk functions $g_x$ and $g_{x'}$ are now *N*-dimensional vectors and that $g_x(x_n)$ is the *n*-th coordinate of $g_x$.

The cross-spectrum of the reconstructed field is

$$\begin{aligned} \hat{\gamma }(x,x') = \sum _{n=1}^N\sum _{m=1}^Ng_x(x_n)g_{x'}(x_m)\gamma _{n,m}, \end{aligned}$$

which can be decomposed as

18 $$\begin{aligned} \hat{\gamma }(x,x') = \sum _{k=1}^Ng_x(x_k)g_{x'}(x_k)\sigma _k^2 + \sum _{n\ne m}g_x(x_n)g_{x'}(x_m)\text {Re}(\gamma _{n,m}) + i \sum _{n\ne m}g_x(x_n)g_{x'}(x_m)\text {Im}(\gamma _{n,m}) \end{aligned}$$

where $\sigma _k^2 = \gamma _{k,k}$ is the power of the *k*-th source. Equation (18) is the discrete version of Eq. (5). Note that the first term (zeroth-order mixing) is independent of the interaction structure of the sources and only depends on their power, whereas the second and third terms (first- and second-order mixing, respectively) only depend on the instantaneous and lagged interaction structure, respectively, and are independent of power. The basic identities from Sect. 2.4 take the form

19 $$\begin{aligned} \text {Re}(\hat{\gamma }(x,x')) = \sum _{n<m}(g_x(x_n)g_{x'}(x_m) + g_{x'}(x_n)g_x(x_m))\text {Re}(\gamma _{n,m}), \end{aligned}$$

and

20 $$\begin{aligned} \text {Im}(\hat{\gamma }(x,x')) = \sum _{n<m}(g_x(x_n)g_{x'}(x_m) - g_{x'}(x_n)g_x(x_m))\text {Im}(\gamma _{n,m}). \end{aligned}$$

The term $||g_x\wedge g_{x'}||^2$ reduces to

21 $$\begin{aligned} ||g_x\wedge g_{x'}||^2 = \sum _{n<m}\left( g_x(x_n)g_{x'}(x_m) - g_{x'}(x_n)g_x(x_m) \right) ^2, \end{aligned}$$

hence the notation $g_x\wedge g_{x'}$ now gets meaning because it is equal to the *wedge product* between the vectors $g_x$ and $g_{x'}$, which is defined as

22 $$\begin{aligned} g_x\wedge g_{x'} = (g_x(x_1)g_{x'}(x_2) - g_{x'}(x_1)g_x(x_2),\dots ,g_x(x_{p-1})g_{x'}(x_p) - g_{x'}(x_{p-1})g_x(x_p)), \end{aligned}$$

and is a vector of dimension $N(N-1)/2$. Strictly speaking, the wedge product is not a vector at all, but an oriented plane spanned by $g_x$ and $g_{x'}$. It is a generalization of the cross product to higher dimensional vectors: In the special case $N=3$, $g_x = (a,b,c)$ and $g_{x'} = (d,e,f)$ are vectors in $\mathbb {R}^3$ and (after permutation of its entries and signs) their wedge product reduces to

$$\begin{aligned} g_x\wedge g_{x'} = (bf-ce,cd-af,ae-bd) = g_x\times g_{x'}, \end{aligned}$$

where $g_x\times g_{x'}$ denotes the cross-product between $g_x$ and $g_{x'}$ and, as such, satisfies the same properties, e.g. $g_x\wedge g_{x} = 0$ and $g_{x'}\wedge g_x = - g_x\wedge g_{x'}$. In fact, the wedge product is uniquely determined by these axioms. Lagrange’s identity for cross-talk functions hence reduces to

23 $$\begin{aligned} ||g_x\times g_{x'}||^2 + \left<g_x,g_{x'}\right> = ||g_x||^2 ||g_{x'}||^2, \end{aligned}$$

and in the normalized case this reduces to Eq. (15).

## Discussion

In this study we established several basic properties regarding the effects of instantaneous linear mixing on the reconstructed cross-spectra of random neural fields. Although these properties are rather superficial from a mathematical point of view, they do provide some insight into aspects of signal mixing that are relevant to experimentalists working with EEG or MEG data. For instance, one of the results is that second-order mixing effects are most severe when the measurement locations are far apart and the sources are located in the vicinity of the measurement locations. The result provides some formal understanding of the large number of false positive interactions in the vicinity of the measurement locations as observed via numerical simulations (Palva et al. 2018).

Although mixing effects are usually studied within a discrete framework by discretizing the source space, we used continuous kernels and random neural fields on cortical manifolds, because it allows for a more natural description of some of the effects, e.g. the relation between mixing effects and the curvature of the point-spread functions. Another reason for adopting this framework is that macroscopic cortical activity is a spatiotemporal phenomenon exhibiting properties such as traveling waves, which are more naturally studied within such a framework. For instance, a description of mixing effects in the spatial frequency domain is readily obtained from the continuous description used in this study by taking the spatial Fourier transforms of the neural fields and the resolution kernels (Hindriks 2020b).

An obvious next question is how the coherences (i.e. normalized cross-spectra) of the true and reconstructed fields are related. Although the decomposition into zeroth-, first-, and second-order effects (see Eq. (5)) is still valid, the coherence is a non-linear function of the different terms and this considerably complicates the analysis. One faces similar difficulties when analyzing the relationship between non-linear properties of the true and reconstructed fields, for example their phase- or amplitude-dynamics. For example, in Hindriks et al. (2016) forward simulations are used to explore the highly non-linear relation between true and observed phase-fields in the context of local field potential recordings. One of the effects that could be analyzed mathematically is phase-contraction, which refers to the fact that the phase-difference between reconstructed signals is typically smaller than that between the true signals, which leads, for example, to overestimation of propagation speeds of neural activity.

The resolution operator was modeled by a linear integral operator with general real-valued kernel and some simple choices for the kernel were considered (e.g. a Gaussian kernel). Although this allowed to clarify some basic aspects of second-order signal leakage, the study of specific effects requires making *ad hoc* choices. For example, effects of source depth can be incorporated by suitably parametrizing the kernel, e.g. by multiplying it with a positive constant $<1$ and increasing its spatial width. These choices, however, are not derived from first principles. To arrive at a more fundamental formalism, the forward and inverse operators that make up the resolution operator should be modelled explicitly. For MEG, the forward operator is given by the Àmpere–Laplace law, and for EEG and ECoG, the forward operator is given by the integral form of Poisson’s equation, both of which are linear integral operators (Hämäläinen et al. 1993). Effects of source depth, unknown dipole orientation, etc. can then be studied from first principles without the need for *ad hoc* choices. Since the brain is modeled as a spatial continuum, it does, however, require the use of inverse operators that map sensor activity into a Hilbert space of brain activity (in contrast to a finite-dimensional vector space). In contrast to the field of electromagnetic brain imaging (Grech et al. 2008), such continuous formulations of inverse methods are standard in most other fields, e.g. acoustic scattering, optical tomography, and seismology, and enable rigorous mathematical analysis (for instance, see (Zhdanov 2002)).

## Data Availability

Data sharing is not applicable to this article as no datasets were generated or analysed during the current study.

## Appendices

### Appendix A: Derivation of the Basic Identities

The above formulas can be derived in the following way. Let $\Omega = \mathbb {R}^2$ so that for $x,x'\in \mathbb {R}^2$:

$$\begin{aligned} \hat{\gamma }(x,x') = \int _{\mathbb {R}^4}g_x(y)g_{x'}(z)\gamma (y,z), \end{aligned}$$

where $y=(y_1,y_2)$ and $z=(z_1,z_2)$ are in $\mathbb {R}^2$. Define subsets of $\mathbb {R}^4$ by $A^+ = \{(y_1,y_2,z_1,z_2) | y_1 > z_1\}$ and $A^- = \{(y_1,y_2,z_1,z_2) | y_1 < z_1\}$ and write

$$\begin{aligned} \hat{\gamma }(x,x') = \int _{A^+}g_x(y)g_{x'}(z)\gamma (y,z) + \int _{A^-}g_{x}(y)g_{x'}(z)\gamma (y,z). \end{aligned}$$

Now define $f:\mathbb {R}^4\rightarrow \mathbb {R}^4$ by $f(x,y) = (y,x)$ and note that *f* induces a diffeomorphism between $A^+$ and $A^-$ with (absolute value of) determinant 1 so that we can write the second term of the above equation as:

$$\begin{aligned} \int _{A^-}g_x(y)g_{x'}(z)\gamma (y,z) = \int _{A^+}g_x(z)g_{x'}(y)\gamma (z,y) = \int _{A^+}g_x(z)g_{x'}(y)\gamma (y,z)^*, \end{aligned}$$

where in the last step we have used the conjugate symmetry of $\gamma$, i.e. $\gamma (z,y) = \gamma (y,z)^*$ for all $x,y\in \mathbb {R}^2$. We can thus rewrite $\hat{\gamma }(x,x')$ as an integral over $A^+$:

$$\begin{aligned} \hat{\gamma }(x,x') = \int _{A^+}\left( g_x(y)g_{x'}(z)\gamma (y,z) + g_x(z)g_{x'}(y)\gamma (y,z)^*\right) , \end{aligned}$$

and therefore its real and imaginary parts are

$$\begin{aligned} \text {Re}(\hat{\gamma }(x,x')) = \int _{A^+}\left( g_x(y)g_{x'}(z) + g_{x'}(y)g_x(z)\right) \text {Re}(\gamma (y,z)), \end{aligned}$$

and

$$\begin{aligned} \text {Im}(\hat{\gamma }(x,x')) = \int _{A^+}\left( g_x(y)g_{x'}(z) - g_{x'}(y)g_x(z)\right) \text {Im}(\gamma (y,z)). \end{aligned}$$

### Appendix B: Local Approximation of Second-Order Leakage

We consider the configuration $(x,x',y,z)$, where *x* and $x'$ are measurement locations and *y* and *z* are source locations and assume that *y* is close to *x* and *z* is close to $x'$. We also assume that, for all locations *v*, the cross-talk function $g_v(u)$ is maximal at $u=v$ and decreases with increasing Euclidean distance between *u* and *v*. Under these assumptions, $g_{x'}(y)g_x(z)\approx 0$ so that $S_-(x,x',y,z)$ and $S_+(x,x',y,z)$ are approximately equal to $g_x(y)g_{x'}(z)$. Since *y* is close to *x* and *z* is close to $x'$, $g_x(y)$ and $g_{x'}(z)$ can be approximated by second-order Taylor series around *x* and $x'$, respectively:

$$\begin{aligned} g_x(y) \approx g_x(x) + \frac{1}{2}(y-x)^\textrm{T}H_g(x)(y-x), \end{aligned}$$

and

$$\begin{aligned} g_{x'}(z) \approx g_{x'}(x') + \frac{1}{2}(z-x')^\textrm{T}H_g(x')(z-x'), \end{aligned}$$

where $H_g(x)$ and $H_g(x')$ denote the Hessian matrices of $g_x$ at *x* and of $g_{x'}$ at $x'$, respectively. Thus, the (*i*, *j*)-th entries of $H_g(x)$ and $H_g(x')$ are equal to $\partial ^2 g_x(x)/\partial x_ix_j,$ and $\partial ^2 g_{x'}(x')/\partial {x'}_i{x'}_j,$ respectively. The first-order terms in the Taylor series of $g_x$ and $g_{x'}$ vanish due to the assumptions that $g_x$ and $g_{x'}$ are maximal at *x* and $x'$, respectively. Combining the approximations for $g_x(y)$ and $g_{x'}(z)$ yields the following approximation for $S_-(x,x',y,z)$:

$$\begin{aligned} S_-(x,x',y,z)\approx & {} g_x(x)g_{x'}(x') + \frac{1}{2}(y-x)^\textrm{T}H_g(x)(y-x)g_{x'}(x') + \frac{1}{2}(z-x')^\textrm{T}H_g(x')(z-x')g_{x}(x)\\ {}+ & {} \frac{1}{4}(y-x)^\textrm{T}H_g(x)(y-x)(z-x')^\textrm{T}H_g(x')(z-x'), \end{aligned}$$

which is also valid for $S_+(x,x',y,z)$. If *y* is sufficiently close to *x* and *z* is sufficiently close to $x'$, the last term on the right-hand side will be much smaller than the second- and third terms, thus we can further approximate $S_+(x,x',y,z)$ as

$$\begin{aligned} S_-(x,x',y,z) \approx g_x(x)g_{x'}(x') + \frac{1}{2}(y-x)^\textrm{T}H_g(x)(y-x)g_{x'}(x') + \frac{1}{2}(z-x')^\textrm{T}H_g(x')(z-x')g_{x}(x), \end{aligned}$$

which is also valid for $S_+(x,x',y,z)$.

### Appendix C: Derivation of Lagrange’s Identity

To prove Lagrange’s identity, we again use the diffeomorphism *g* between $A^+$ and $A^-$ to write

$$\begin{aligned} ||g_x\wedge g_{x'}||^2= & {} \frac{1}{2}\int _{\mathbb {R}^4}\left( g_x(y)g_{x'}(z) - g_{x'}(y)g_x(z)\right) ^2 \\ {}{} & {} \\ {}= & {} \frac{1}{2}\int _{\mathbb {R}^4}\left( g_x(y)^2g_{x'}(z)^2 + g_{x'}(y)^2g_x(z)^2 - 2g_x(y)g_{x'}(z)g_{x'}(y)g_x(z)\right) \\ {}{} & {} \\= & {} \int _{\mathbb {R}^4}g_x(y)^2g_{x'}(z)^2 - \int _{\mathbb {R}^4}g_x(y)g_{x'}(y)\int _{\mathbb {R}^4}g_{x}(z)g_{x'}(z)\\{} & {} \\ {}= & {} ||g_x||^2 ||g_{x'}||^2 - \left<g_x,g_{x'}\right>^2. \end{aligned}$$
